# Development of an application for colorectal cancer prediction using SDC2 and TFPI2 methylation: an opportunity for early and noninvasive diagnosis in Latin-America

**DOI:** 10.3389/fmolb.2026.1922606

**Published:** 2026-09-22

**Authors:** Fernando Hernandez-Medina, Alejandro Cornejo, Andrea Orue

**Affiliations:** 1 Laboratorio de Inmunopatología, Centro de Medicina Experimental, Instituto Venezolano de Investigaciones Científicas. IVIC, Caracas, Venezuela; 2 Laboratorio de Biología de Tumores, Centro de Microbiología y Biología Celular, Instituto Venezolano de Investigaciones Científicas. IVIC, Caracas, Venezuela

**Keywords:** application technology, biomarker-methylation, colorectal-cancer, early-detection, Latin-America, SDC2, TFPI2

## Abstract

Colorectal cancer remains a major public health challenge globally and within Latin-America, where expanding mortality rates are compounded by heterogeneous access to screening programs, healthcare infrastructure. While early detection is critical to improving survival rates, conventional fecal tests and colonoscopies face significant financial, logistical, and patient-adherence barriers in resource-limited settings. Epigenetic alterations, specifically promoter hypermethylation of CpG islands, occur early during the adenoma–carcinoma sequence and provide stable, highly sensitive targets for noninvasive molecular diagnostics. In this Perspective, we discuss the molecular rationale and complementary diagnostic value of combining two robustly validated epigenetic biomarkers, Syndecan-2 (SDC2), which modulates epithelial integrity and signaling, and Tissue Factor Pathway Inhibitor-2 (TFPI2), a critical serine protease inhibitor regulating extracellular matrix remodeling. Transcriptional silencing of these genes through aberrant hypermethylation reflects distinct yet commentary pathways of colorectal carcinogenesis, providing the molecular foundation for the implementation strategy proposed herein. To overcome technical barriers that currently limit the broader adoption of methylation-based screening, we present the integration of validated quantitative real-time PCR (qPCR) methylation assays with ColoGuardIA, an open-source, lightweight, Shiny-based digital application developed by our group as an implementation-oriented proof-of-concept platform. ColoGuardIA facilitates the standardized interpretation of qPCR-derived cycle threshold (Ct) values generated by previously validated methylation tests. By helping bridge the gap between molecular oncology and decentralized clinical implementation, this framework represents a scalable strategy to support the adoption of noninvasive methylation-based screening approaches and improve access to early colorectal cancer detection in Latin-America.

## 1 Introduction

Colorectal cancer (CRC) remains one of the most significant global public health challenges, currently ranking as the third most commonly diagnosed malignancy and the second leading cause of cancer-related death worldwide (Rabeneck et al., 2020; Bray et al., 2024). According to GLOBOCAN estimates, more than 1.9 million new CRC cases and approximately 930,000 deaths were reported in 2020, with these numbers projected to increase substantially over the coming decades (GBD, 2019). Although historically CRC incidence and mortality have been higher in high-income countries, the burden of disease is rising rapidly across low- and middle-income regions, including Latin-America (Arnold et al., 2017; Keum and Giovannucci, 2019). Here, demographic transitions, aging populations, dietary shifts, sedentary lifestyles, and the increasing consumption of ultra-processed foods drive a sharp increase in CRC cases (Matos et al., 2021). This epidemiological transition poses a challenge for Latin-American healthcare systems (Demb and Gupta, 2020). Despite advances in oncology, access to organized screening programs remains highly heterogeneous across the region, and substantial disparities persist in healthcare infrastructure, specialist availability, and access to diagnostic technologies (Barrios et al., 2021; Nuche-Berenguer and Sakellariou, 2019; Muzi et al., 2023). Consequently, a vast majority of CRC cases continue to be diagnosed at advanced stages, drastically reducing treatment efficacy and driving up mortality rates.

Importantly, CRC develops through a prolonged, multistep process involving the accumulation of genetic and epigenetic alterations that transform normal colonic epithelium into adenomatous lesions and eventually invasive carcinoma (Dekker et al., 2019). This adenoma–carcinoma sequence frequently extends over 10–15 years, providing a critical window for early detection and intervention (Han et al., 2019; Cao el, 2024). The multistep progression from normal epithelium to invasive carcinoma involves not only genetic and epigenetic alterations, but also dysregulation of key oncogenic signaling pathways such as PI3K/AKT/mTOR, which contribute to colorectal tumorigenesis (Tong et al., 2022). Early-stage detection is associated with five-year survival rates exceeding 90%, whereas metastatic disease drops outcomes below 15% (Rezkitha et al., 2024; Kautto et al., 2017).

Current screening strategies, including colonoscopy, flexible sigmoidoscopy, fecal immunochemical testing (FIT), and guaiac fecal occult blood testing (gFOBT), have significantly reduced CRC mortality where large-scale screening is successfully implemented (Ladabaum et al., 2019; Han et al., 2019). However, widespread adoption of these approaches remains challenging in many Latin-American countries due to financial constraints, limited healthcare infrastructure, insufficient specialist coverage, and low patient adherence to invasive procedures (Calderon-Aparicio and Orue, 2019; Montalvan-Sanchez et al., 2024).

In recent years, molecular diagnostics have emerged as promising non-invasive alternatives (Cervena et al., 2020; Song et al., 2023). Among these, DNA methylation biomarkers have attracted considerable attention because promoter hypermethylation represents an early, frequent, and stable event during colorectal carcinogenesis that can be detected non-invasively in stool-derived DNA (Bray et al., 2024; Qi et al., 2026. The promoter methylation of Syndecan-2 (SDC2) and Tissue Factor Pathway Inhibitor-2 (TFPI2) has demonstrated high sensitivity and specificity for detecting both CRC and advanced precancerous lesions across multiple populations (Lei et al., 2022; Wang et al., 2025). Given the growing evidence supporting the clinical utility of SDC2 and TFPI2 methylation assays, there is an urgent need to facilitate their implementation in resource-constrained settings. In this Perspective, we discuss the molecular rationale and published clinical evidence supporting the use of SDC2 and TFPI2 methylation as early CRC biomarkers and propose their integration with ColoGuardIA, a lightweight, open-source, Shiny-based digital application developed by our group as an implementation-oriented proof-of-concept platform. This platform is designed to facilitate the standardized interpretation of methylation assay results generated by laboratory, bridging the gap between molecular diagnostics and clinical actionability in low-resource Latin-American healthcare environments.

## 2 Epigenetic basis of early colorectal cancer detection

Colorectal carcinogenesis is driven by the progressive accumulation of both genetic and epigenetic alterations (Kuipers et al., 2015). While genetic mutations affecting genes such as *APC*, *KRAS*, *PIK3CA*, and *TP53* are established drivers, epigenetic alterations are equally critical contributors to disease initiation (Sung et al., 2021; Bray et al., 2024). Among these, DNA methylation—the addition of a methyl group to cytosine residues within CpG dinucleotides catalyzed by DNA methyltransferases—is the most extensively studied and clinically relevant alteration in CRC (Cao et al., 2024). Under physiological conditions, methylation regulates gene expression, maintains genomic stability, and directs cellular differentiation (Cao et al., 2024). However, aberrant methylation disrupts normal cellular homeostasis during tumorigenesis (Bray et al., 2024).

A hallmark of CRC is the hypermethylation of CpG-rich promoter regions (CpG islands), leading to transcriptional silencing of genes involved in cell-cycle regulation, DNA repair, apoptosis, cell adhesion, and tumor suppression (Bray et al., 2024; Sung et al., 2021). Unlike many genetic mutations that accumulate later during disease progression, promoter hypermethylation often occurs at the earliest stages of neoplastic transformation, including premalignant adenomas and sessile serrated lesions, making it an ideal target for early screening (Kuipers et al., 2015; Rezkitha et al., 2024). One of the major molecular pathways associated with colorectal carcinogenesis is the CpG Island Methylator Phenotype (CIMP), characterized by widespread promoter hypermethylation affecting multiple genes simultaneously. CIMP-positive tumors frequently exhibit epigenetic silencing of critical regulatory genes and are often associated with the serrated neoplasia pathway. Although colorectal cancer has traditionally been classified into chromosomal instability (CIN), microsatellite instability (MSI), and CIMP pathways, growing evidence suggests substantial biological overlap among these mechanisms, highlighting the central role of epigenetic dysregulation in disease evolution (Dekker et al., 2019; Cervena et al., 2020; Bray et al., 2024). Beyond tumor-cell-intrinsic genetic and epigenetic events, the immune and inflammatory microenvironment can substantially influence disease progression and biological heterogeneity across digestive tract disorders (Wang et al., 2023).

Furthermore, aberrantly methylated DNA is released into biological specimens, including stool and plasma, where it remains sufficiently stable for molecular detection using methylation-specific PCR-based approaches (Lei et al., 2022). Compared with mutation-based biomarkers, methylation markers are often more prevalent and detectable across a broader spectrum of early-stage lesions, increasing their utility for population-based screening (Han et al., 2019). Among the numerous candidates evaluated to date, SDC2 and TFPI2 have emerged as two of the most consistently validated biomarkers, demonstrating robust performance across multiple populations and disease stages.

## 3 Molecular rationale for SDC2 and TFPI2 methylation biomarkers

The principal analytical characteristics and diagnostic performance reported for SDC2 and TFPI2 methylation assays across representative clinical studies are summarized in Supplementary Table S1.

### 3.1 SDC2 methylation as an early biomarker of colorectal carcinogenesis

Syndecan-2 (SDC2) is a transmembrane heparan sulfate proteoglycan that plays a central role in cell–cell and cell–extracellular matrix interactions. Physiologically, SDC2 contributes to epithelial integrity, cellular adhesion, migration, cytoskeletal organization, and tissue homeostasis, including modulation of Wnt/β-catenin signaling (Han et al., 2019). These functions are relevant in the intestinal epithelium, where controlled regulation of proliferation is essential for maintaining normal crypt architecture. During colorectal tumorigenesis, aberrant promoter hypermethylation of SDC2 leads to transcriptional silencing and disruption of its regulatory functions. This epigenetic alteration has been consistently detected in early colorectal adenomas, sessile serrated lesions, and invasive carcinomas (Han et al., 2019; Lei et al., 2022). Clinical studies have demonstrated the diagnostic value of stool-based SDC2 methylation (Oh et al., 2017), with sensitivities approaching 90% for CRC detection, including excellent performance in asymptomatic stage I and II disease (Bagheri et al., 2020).

### 3.2 TFPI2 methylation and loss of tumor suppressor function

Tissue Factor Pathway Inhibitor-2 (TFPI2) is a Kunitz-type serine protease inhibitor that regulates extracellular matrix remodeling and tissue homeostasis. Physiologically, TFPI2 suppresses excessive proteolytic activity, limiting the degradation of extracellular matrix components and preventing inappropriate cellular invasion (Wang et al., 2025). Through these mechanisms, TFPI2 acts as a potent tumor suppressor. In CRC, TFPI2 promoter hypermethylation results in transcriptional silencing and loss of its protective anti-invasive functions early during neoplastic progression (Lei et al., 2022; Wang et al., 2025). Functional studies show that restoring TFPI2 expression suppresses cellular proliferation, migration, and invasion. From a diagnostic perspective, a recent meta-analysis reported pooled sensitivity and specificity values of approximately 83% and 96%, respectively, with an area under the receiver operating characteristic curve approaching 0.97, highlighting its robustness as an independent biomarker (Wang et al., 2025).

### 3.3 Complementary diagnostic value of combined SDC2 and TFPI2 detection

Evidence suggests that the combined assessment of SDC2 and TFPI2 provides superior sensitivity and robustness compared with either biomarker alone (Lei et al., 2022). From a biological perspective, this improvement is highly plausible because the two genes participate in distinct, complementary aspects of colorectal carcinogenesis. While SDC2 methylation reflects alterations in epithelial homeostasis and signaling pathways associated with early neoplastic transformation, TFPI2 methylation captures the loss of extracellular matrix regulation and anti-invasive tumor suppressor activity.

Simultaneous evaluation of both biomarkers captures a broader spectrum of molecular alterations occurring across the adenoma–carcinoma sequence. Multicenter studies have reported that combined SDC2/TFPI2 methylation panels achieve sensitivities exceeding 85% for early-stage CRC while maintaining high specificity (Bagheri et al., 2020). The molecular cascade of epigenetic dysregulation and subsequent gene silencing is schematized in Figure 1. While physiological unmethylated CpG islands allow normal gene expression, aberrant hypermethylation mediated by DNMTs triggers chromatin condensation and transcriptional silencing (Figure 1A). Pathologically, SDC2 silencing disrupts epithelial crypt homeostasis, whereas TFPI2 inactivation leads to unchecked proteolytic degradation of the extracellular matrix, cooperatively driving progression from early adenomas to invasive carcinomas (Figure 1B). Crucially, the combined approach improves the detection of advanced adenomas and sessile serrated lesions—lesions that are frequently missed by conventional fecal occult blood tests—positioning this dual panel among the most promising non-invasive molecular tools available. The integration of diverse molecular data, including methylation profiles, transcriptomic features, microbiome signatures, and histopathological information, is increasingly recognized as an important strategy for improving risk stratification and personalized management in cancer (He et al., 2026).

**FIGURE 1:**
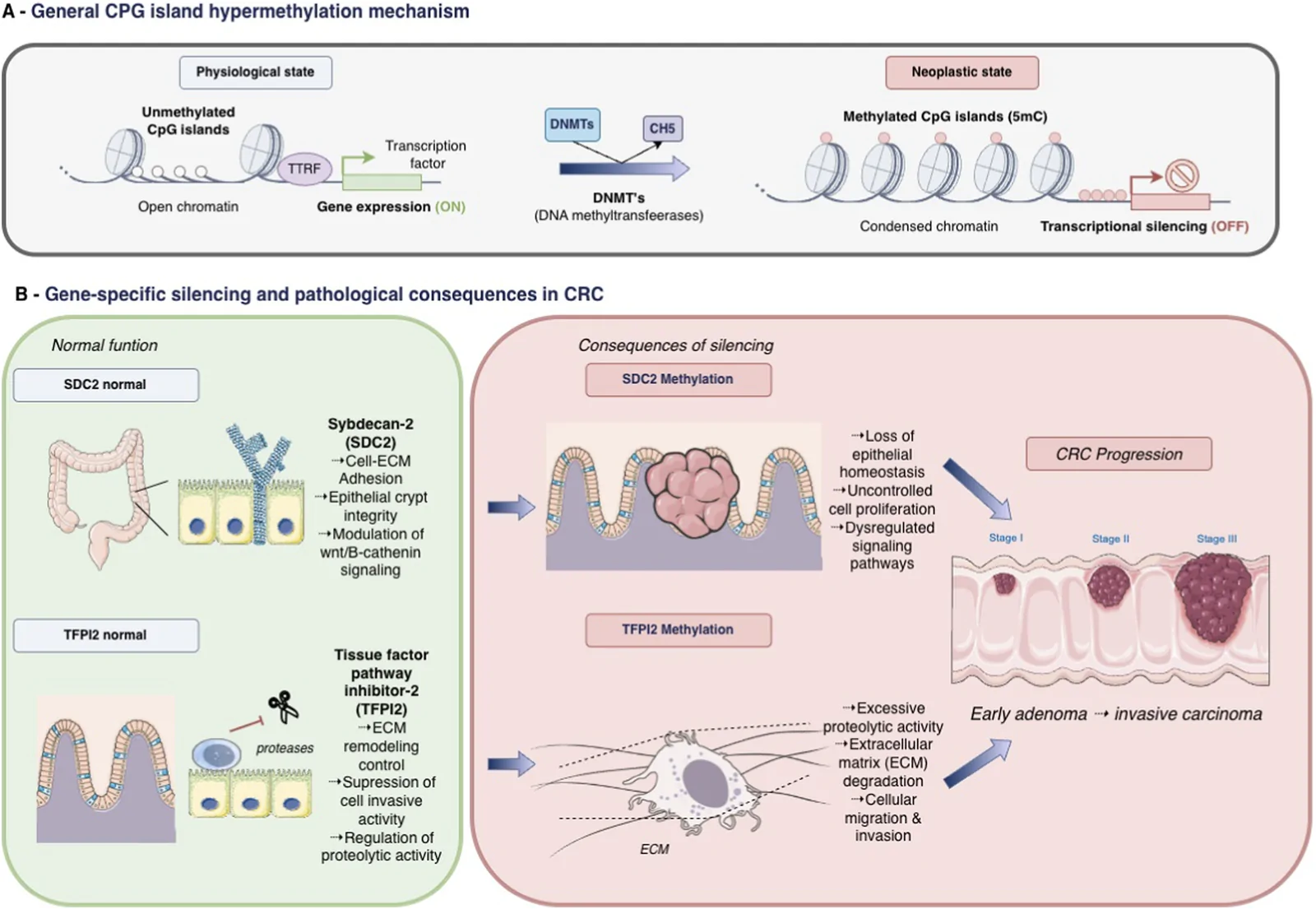
Molecular pathways of epigenetic dysregulation and gene-specific silencing in colorectal carcinogenesis. **(A)** Schematic representation of CpG island hypermethylation. In the physiological-condition, open chromatin architecture allows transcription factor recruitment and active gene expression (ON). In the neoplastic condition, DNA methyltransferases (DNMTs) induce hypermethylation (5 mC), leading to condensed chromatin and transcriptional silencing (OFF). **(B)** Biological consequences of specific biomarker silencing. SDC2 hypermethylation triggers the loss of epithelial crypt integrity and altered signaling pathways. TFPI2 hypermethylation leads to uncontrolled protease activity, extracellular matrix (ECM) degradation, and cellular invasion, cooperatively driving progression from early adenoma to invasive carcinoma.

## 4 Latin-America and accessible molecular screening strategies

Despite the evidence supporting methylation-based biomarkers for colorectal cancer detection, their incorporation into routine screening programs remains limited throughout Latin-America (Bamodu and Chung, 2024). This gap reflects severe socioeconomic and infrastructural disparities between healthcare systems of the region and the difficulties associated with translating molecular discoveries into practical clinical tools (Sanguinetti et al., 2020; Montalvan-Sanchez et al., 2024). While colonoscopy remains the gold standard for colorectal cancer detection, its implementation requires specialized personnel, endoscopic facilities, bowel preparation, and substantial financial investment, creating important barriers to widespread adoption (Ladabaum et al., 2019; Calderon-Aparicio and Orue, 2019). Alongside molecular biomarkers, advances in computational pathology and deep learning are providing new opportunities to predict tumor characteristics and risk, thereby complementing specimen-based molecular tests with image-derived diagnostic information (Zhang et al., 2025).

Furthermore, access to molecular diagnostics is deeply unequal. In many low-resource Latin-American settings, local or regional laboratories possess the basic equipment required to perform real-time PCR assays, yet they completely lack bioinformatic support, specialized molecular pathology personnel, or standardized criteria to interpret the resulting raw data. This lack of translational tools means that even when a laboratory manages to run a methylation assay, the raw outputs cannot be easily translated into a clear clinical decision by a primary care physician. While molecular screening can identify individuals at increased risk, accurate preoperative local staging using imaging modalities such as MRI and endorectal ultrasound remains essential for treatment planning and optimal clinical management in rectal cancer (Yang et al., 2025). Therefore, the next crucial stage in molecular diagnostics for low-resource environments is not the discovery of new biomarkers, but the development of accessible, user-friendly digital tools capable of standardizing interpretation across diverse, fragmented healthcare environments. While commercial assays based on SDC2 and TFPI2 have shown excellent performance in Asian and high-income populations (Han et al., 2019; Lei et al., 2022), their implementation in Latin-America will require simple, reproducible, and easily interpretable workflows that can be deployed across laboratories with different levels of technical expertise.

## 5 Facilitating clinical implementation in resource-constrained settings: ColoGuardIA

### 5.1 From molecular biomarkers to digital democratization

The successful translation of SDC2 and TFPI2 from bench to bedside depends heavily on the availability of standardized, accessible workflows (Lei et al., 2022; Wang et al., 2025). Although commercial assays exist, their high costs and proprietary interpretation algorithms make them inaccessible for public health systems in Latin-America. Interpretation of methylation parameters can vary widely between institutions and operators, a challenge amplified in low-resource settings where access to specialized bioinformaticians is limited (Hendl and Shukla, 2024). Consequently, there is a growing need for practical tools that facilitate result interpretation while preserving the biological significance of these validated biomarkers (Yin et al., 2023). To bridge this gap, digital health democratization offers a viable solution (The Lancet Digital Health, 2021). By leveraging open-source software, molecular assay outputs can be transformed into a unified, easy-to-read, and standardized interpretation report that facilitates communication between molecular laboratories and healthcare professionals. This approach aims to standardize result interpretation across laboratories, helping reduce variability in reporting independently of geographical location or available technical expertise.

### 5.2 Detection methodology and interpretation challenges

The detection of SDC2 and TFPI2 methylation typically relies on DNA extracted from stool or plasma, followed by sodium bisulfite conversion to discriminate between methylated and unmethylated cytosines via methylation-specific PCR (MSP) or quantitative real-time PCR (qPCR) (Rezkitha et al., 2024; Khabbazpour et al., 2025; Ma et al., 2022). In clinical practice, the outputs of these assays are represented by amplification curves, cycle threshold (Ct) values, methylation percentages or qualitative methylation calls. While these parameters are routine for specialized molecular laboratories, they are often difficult to interpret for general clinicians and healthcare professionals leading field screening programs. Variations in reporting formats further complicate integration into routine clinical workflows, emphasizing the need for tools that simplify and unify data interpretation without losing the underlying biological significance. Accordingly, implementation-oriented digital tools should be viewed as complementary resources that facilitate standardized interpretation of validated laboratory results rather than as substitutes for molecular testing or clinical decision-making.

### 5.3 ColoGuardIA: an open-source digital support platform

To address these implementation challenges, we developed ColoGuardIA (https://github.com/fernandoh76/ColoGuardIA; and https://fernandoh76ve.shinyapps.io/shinyappproject/), a lightweight Shiny-based application designed to facilitate the standardized interpretation of SDC2 and TFPI2 methylation results. Rather than functioning as a diagnostic assay, ColoGuardIA serves as a digital support platform that translates molecular outputs into an accessible and standardized format. The current implementation-oriented proof-of-concept implementation accepts methylation results generated by validated methylation-specific qPCR assays. Support for microarray-based platforms and next-generation sequencing (NGS) methylation analyses will be incorporated in a future update. Using predefined interpretation criteria derived from published evidence (Lei et al., 2022), the platform provides an intuitive interface for visualizing methylation status and generating standardized reports that may facilitate communication between molecular laboratories and clinicians. In its initial deployment, application usage requires DNA methylation data obtained via specific qPCR assays for the SDC2 and TFPI2 genes. The application compares user-uploaded qPCR-derived methylation results with predefined reference thresholds obtained from published studies, thereby facilitating standardized result interpretation.

The translational pipeline from biological sampling to automated digital reporting is structured as an integrated workflow in Figure 2. Following noninvasive sample collection, bisulfite conversion, and qPCR analysis for SDC2 and TFPI2, raw cycle threshold (Ct) values are captured (Figure 2 Steps 1–6). These molecular outputs are ingested by ColoGuardIA, which leverages an Isolation Forest anomaly detection model for automated quality control to deliver standardized clinical risk profiles and subsequent colonoscopy triage recommendations (Figure 2 Steps 7–9). Importantly, ColoGuardIA does not replace molecular testing nor clinical judgment. Instead, it aims to reduce interpretation variability, improve result standardization, and facilitate the incorporation of methylation biomarkers into routine screening workflows. By simplifying the interpretation process, the platform may contribute to broader adoption of molecular screening approaches in healthcare environments where specialized expertise is limited.

**FIGURE 2:**
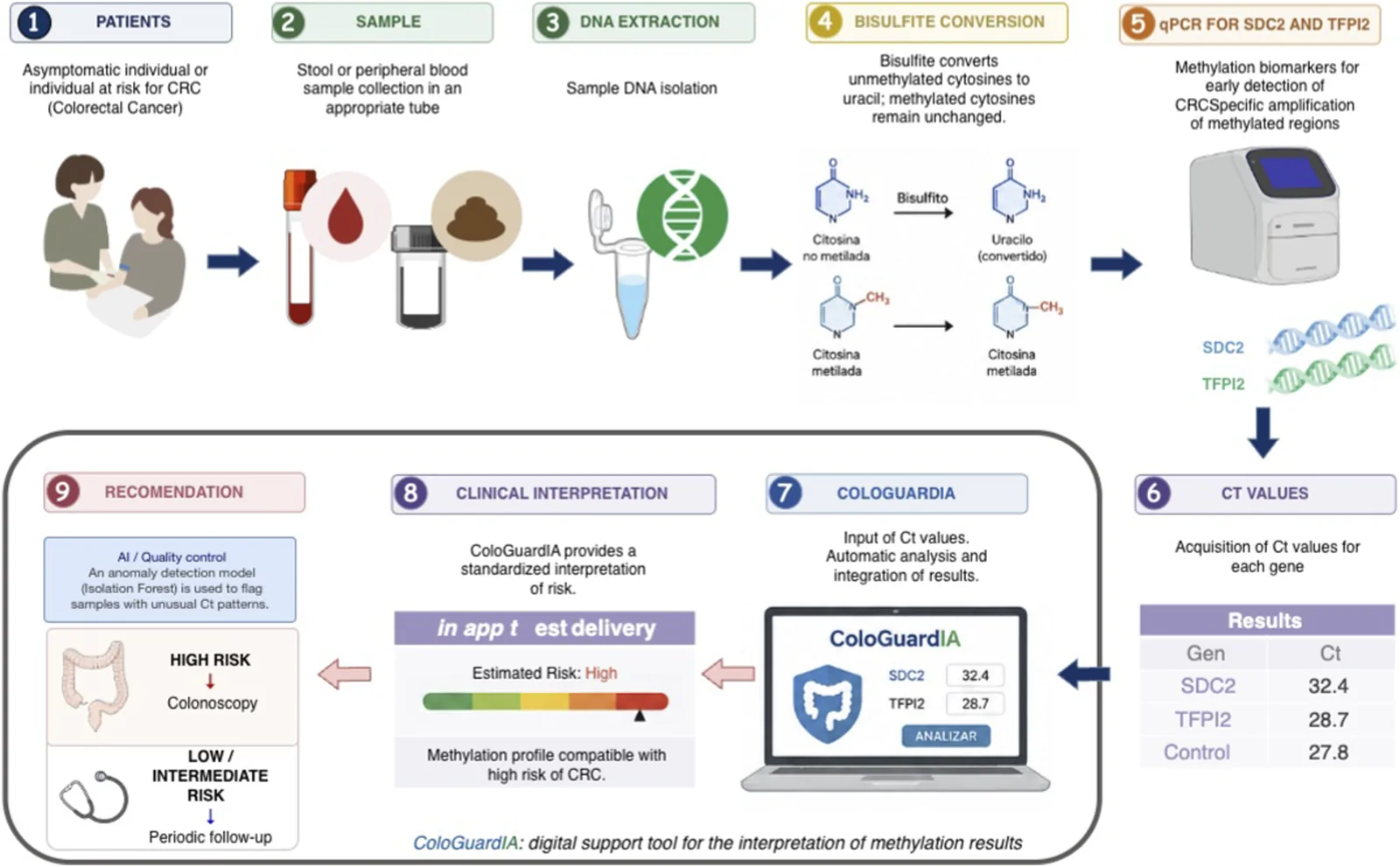
Translational workflow for noninvasive colorectal cancer screening integrated with the ColoGuardIA digital support platform. The operational schematic depicts the sequential steps from patient stratification to clinical recommendations: (1) identification of candidate individuals; (2) biological specimen collection (stool/blood); (3) DNA isolation; (4) chemical bisulfite conversion; (5) gene-specific qPCR amplification targeting SDC2 and TFPI2 regions; (6) acquisition of experimental cycle threshold (Ct) values; (7) input of molecular metrics into the ColoGuardIA interface; (8) standardized algorithmic risk calculation; and (9) automated clinical triage recommendations incorporating machine learning-based (Isolation Forest) quality control.

### 5.4 Potential relevance for Latin-America

The implementation of molecular diagnostics in Latin-America faces unique challenges related to healthcare inequalities, limited laboratory infrastructure, unequal distribution of specialists, and restricted access to advanced diagnostic technologies and high healthcare costs (Calderon-Aparicio and Orue, 2019; Sanguinetti et al., 2020; Sierra et al., 2016). While methylation-based assays represent a promising opportunity for non-invasive colorectal cancer screening, their widespread adoption requires practical solutions that facilitate their use beyond highly specialized academic centers (Zhang L. et al., 2021; Zhang W. et al., 2021). In this context, implementation-oriented digital tools such as ColoGuardIA may represent a useful translational bridge between molecular biomarker discovery and clinical implementation. By providing a user-friendly environment for interpreting validated methylation biomarkers, such platforms could help standardize reporting practices, facilitate training, support multicenter screening initiatives, and ultimately contribute to expanding access to precision diagnostics throughout the region. Although prospective clinical validation and external evaluation remain necessary before routine clinical adoption, implementation-oriented tools may represent an important first step toward reducing barriers that currently limit the adoption of molecular screening technologies in Latin-America.

## 6 Future perspectives and conclusion

The convergence of validated methylation biomarkers and accessible digital tools offers a unique opportunity to transform CRC screening in Latin-America. However, key milestones must be achieved in the near future. First, generating region-specific clinical evidence is a major priority. Although the diagnostic performance of SDC2 and TFPI2 methylation has been extensively evaluated in Asian, European, and North-American populations, validation studies in Latin-American populations remain limited. Multicenter regional studies are essential to refine the threshold values within the algorithm.

Furthermore, ColoGuardIA is designed to serve as a collaborative repository and could evolve beyond simple interpretation tools and become components of broader precision medicine ecosystems. Future versions could incorporate automated quality-control procedures, longitudinal patient monitoring, integration with electronic medical records, cloud-based multicenter databases for CRC screening, and machine learning algorithms capable of refining risk assessment as additional evidence becomes available. Importantly, these advances should be accompanied by policies that promote equitable access to molecular diagnostics. The successful implementation of precision oncology in Latin-America will depend not only on scientific innovation but also on investments in healthcare infrastructure, workforce training, laboratory capacity, and regional collaborative networks. In this context, implementation-focused technologies may represent a practical strategy for reducing barriers that currently limit access to early colorectal cancer detection.

Ultimately, the convergence of validated methylation biomarkers, accessible molecular technologies, and digital decision-support tools offers a unique opportunity to transform colorectal cancer screening in resource-limited settings. By bridging the gap between biomarker discovery and clinical implementation, these approaches may contribute to more equitable and effective cancer prevention strategies throughout Latin-America.

## Data Availability

The original contributions presented in the study are included in the article/Supplementary Material, further inquiries can be directed to the corresponding author.
