# Supplementary material for: Development of an application for colorectal cancer prediction using SDC2 and TFPI2 methylation: an opportunity for early and noninvasive diagnosis in Latin-America
Source: Front Mol Biosci. 2026 Sep 22;13:1922606. doi: 10.3389/fmolb.2026.1922606 (PMC13639266; doi:10.3389/fmolb.2026.1922606)
Supplement: Supplementary file 1 [file Supplementaryfile1.docx]

# **Supplementary Methods: Isolation Forest Configuration Used in ColoGuardIA**

## **S1. Scope and Intended Use**

The Isolation Forest component in ColoGuardIA is used for **quality control (QC)** by flagging unusual Ct-pattern samples for manual review.
It is implemented as an **unsupervised anomaly detection** step and is not used as a stand-alone diagnostic classifier.

## **S2. Implementation Details**

- Language: **R** (version 5.4.3)
- Framework: **Shiny**
- Anomaly detection library: **solitude**
- Model class: **isolationForest**

Implementation reference:

- app.R (commit: eb8f7e1029fabc7542872ab0b8abb6ccf578b748)

## **S3. Input Features Used by the Model**

The Isolation Forest model is fit on exactly **three numeric features** per sample:

1. Ct_Ref
2. Ct_SDC2
3. Ct_TFPI2

These are extracted in the QC module as:

- X <- df_qc[, c("Ct_Ref", "Ct_SDC2", "Ct_TFPI2")]

## **S4. Preprocessing Applied Before Fitting**

### **S4.1 Type coercion**

Each feature is converted to numeric:

- as.numeric(Ct_Ref)
- as.numeric(Ct_SDC2)
- as.numeric(Ct_TFPI2)

### **S4.2 Missing value handling**

For each feature independently, missing values (NA) are replaced with the **feature median** computed in the QC subset:

- med <- median(X[[nm]], na.rm = TRUE)
- If median is non-finite, fallback is 0
- NA values are then imputed with med

No additional scaling, centering, log transformation, or winsorization is applied in the current implementation.

## **S5. Sample Inclusion Rules for QC**

QC runs on either:

- only samples labeled "Valid" (default/recommended), or
- all processed samples (if user disables that option)

This behavior is controlled by the UI option:

- qc_only_valid (default: TRUE)

Minimum sample size required to run AI QC:

- nrow(df_qc) >= 5

If fewer than 5 samples are available, QC is not executed.

## S6. Isolation Forest Hyperparameters

The model is instantiated as:

- iso <- isolationForest$new(sample_size = min(256, nrow(X)), num_trees = 200, seed = 42)

Therefore, the effective parameters are:

| Parameter | Value | Notes |
| --- | --- | --- |
| sample_size | min(256, nrow(X)) | Caps per-tree subsample at 256; uses all rows when n < 256 |
| num_trees | 200 | Number of isolation trees |
| seed | 42 | Fixed seed for reproducibility |

No additional user-exposed model hyperparameters are defined in the app code.

## **S7. Anomaly Score Computation**

After fitting:

- score <- iso$predict(X)
- sample-level anomaly score: score$anomaly_score
- stored as: df_qc$QC_Score

Higher QC_Score values are treated as more anomalous in downstream ranking.

## **S8. Flagging Threshold and Review Rule**

The app does not use a fixed numeric anomaly-score threshold.
Instead, it uses a **rank-based top-k rule** controlled by the UI contamination slider.

### S8.1 User-set contamination input

- UI parameter: input$contamination
- Range: 0.01 to 0.30
- Default: 0.08
- Step: 0.01

### S8.2 Number of flagged samples

- k <- max(1, floor(nrow(df_qc) * input$contamination))

Thus, at least one sample is always flagged.

### S8.3 Flag assignment

- Sort by decreasing anomaly score:
  - ord <- order(df_qc$QC_Score, decreasing = TRUE)
- Initialize all flags as "OK"
- Assign "Review" to top k scored samples:
  - flag[ord[seq_len(k)]] <- "Review"

Output labels:

- QC_Flag = "OK" or "Review"

## **S9. Related Non-AI Thresholds in the Same Pipeline**

Although not Isolation Forest hyperparameters, the app defines fixed Ct decision thresholds used for primary methylation interpretation:

- ct_ref_SDC2 = 38.0
- ct_ref_TFPI2 = 38.0
- reference_threshold = 36.0 (validity check on Ct_Ref)

These thresholds are applied before/independently of the anomaly detector.

## **S10. Reproducibility Notes**

To reproduce QC outputs exactly, use:

1. The same input data
2. The same preprocessing (numeric coercion + median imputation)
3. The same model settings:
   - sample_size = min(256, nrow(X))
   - num_trees = 200
   - seed = 42
4. The same contamination value (input$contamination)
5. The same inclusion mode (qc_only_valid)

## **S11. Interpretation Caveat**

The Isolation Forest module is designed for **QC triage** (flagging unusual patterns for review), not for independent clinical diagnosis.
Clinical interpretation should remain anchored to validated diagnostic pathways and expert laboratory/clinical judgment.

## **S12. Code Snippet**

iso <- isolationForest$new(
 sample_size = min(256, nrow(X)),
 num_trees = 200,
 seed = 42
)
iso$fit(X)

score <- iso$predict(X)
df_qc$QC_Score <- score$anomaly_score

k <- max(1, floor(nrow(df_qc) * input$contamination))
ord <- order(df_qc$QC_Score, decreasing = TRUE)

flag <- rep(lang()$qc_flag_ok, nrow(df_qc))
flag[ord[seq_len(k)]] <- lang()$qc_flag_review
df_qc$QC_Flag <- flag

### **Abbreviations**

QC, quality control; Ct, cycle threshold.
