# Supplementary material for: Development of an application for colorectal cancer prediction using SDC2 and TFPI2 methylation: an opportunity for early and noninvasive diagnosis in Latin-America
Source: Front Mol Biosci. 2026 Sep 22;13:1922606. doi: 10.3389/fmolb.2026.1922606 (PMC13639266; doi:10.3389/fmolb.2026.1922606)
Supplement: Supplementary file 2 [file Table1.docx]

| **Study (Author, Year)** | **Specimen Type** | **Assay Platform** | **Cutoff Value** | **Biomarker** | **CRC Stage** | **Adenoma Characteristics** | **Sensitivity (%)** | **Specificity (%)** | **AUC** | **95% Confidence Interval (CI)** |
| --- | --- | --- | --- | --- | --- | --- | --- | --- | --- | --- |
| Han et al., 2019 | Stool | meSDC2 LTE-qMSP | Ct < 40 | SDC2 | 0–IV | AA (≥ 1 cm); NA (< 1 cm) | 90.2% | 90.2% | 0.902 | 0.876–0.928 |
| Zhang L. et al., 2021 | Stool | MSP (qMSP) | Ct ≤ 38 | SDC2 / TFPI2 | All stages | 190 mixed adenomas | 96.6% | 96.4% | 0.98 | 0.96–0.99 |
| Zhang W. et al., 2021 | Stool | qMSP | Ct < 38 | SDC2 + TFPI2 | I–IV | AA and NA | 93.4% | 94.3% | NR* | NR |
| Ma et al., 2022 | Stool | qMSP (ColoCaller) | Risk Index > 0.087 | SDC2/TFPI2/ WIF1/NDRG4 | I–IV | AA (> 1 cm) | 95.56% | 91.86% | 0.952 | 85.17–98.77 (Sens) |
| Khabbazpour et al., 2025 | Plasma | MethyQESD | NR | SDC2 + TFPI2 | Polyps only | HRP (≥ 10 mm or > 3 polyps) | 70% | 92% | 0.890 | 0.596–0.843 (AUC) |

**Supplementary Table 1. Systematic Synthesis of Diagnostic Performance Metrics for SDC2 and TFPI2 Methylation in Colorectal Cancer and Precancerous Lesions.**

AUC: Area Under the Curve, AA: Advanced Adenoma, NA: Non-Advanced Adenoma, HRP: High-Risk Polyps, CI: Confidence Interval, qMSP: Quantitative Methylation-Specific Polymerase Chain Reaction, LTE-qMSP: Linear Target Enrichment quantitative Methylation-Specific PCR, MSP: Methylation-Specific PCR, a technique used to identify methylated DNA sequences, NR: Not Reported; indicates that the specific value was not provided in the source study.
